# Integrating Autism and ADHD Assessments Into a Perinatal Mental Health Service: Patient Outcomes and Experiences

**DOI:** 10.1192/bjo.2026.11602

**Published:** 2026-06-30

**Authors:** Shambhavi Pranoy, Katherine Ferrer, Angharad Piette

**Affiliations:** Swansea Bay University Health Board, Swansea, United Kingdom

## Abstract

**Aims:**

To evaluate the implementation of autism and ADHD assessments within the Swansea Bay community perinatal team and mother and baby inpatient unit in South Wales, focusing on service outcomes and patient and staff experiences.

**Methods:**

Perinatal inpatients and outpatients assessed for ADHD or autism from Feb 2024–Aug 2025 were identified. Assessment outcomes were captured, and all 22 patients were invited to provide feedback on experience and impact. MDT members from inpatient andcommunity perinatal services also provided perspectives. Thematic analysis was applied to all responses.

**Results:**

Quantitative: Of 867 screened perinatal cases, 22 underwent assessment (7 inpatients, 15 outpatients). Among inpatients, 6 met diagnostic thresholds (4 autism, 2 ADHD); all 15 outpatients met thresholds (13 autism, 2 ADHD).

Qualitative: Feedback from 12 staff members highlighted that diagnosis improved patient self-advocacy and family relationships but reported gaps in post-diagnostic support, leaving patients to interpret their diagnosis independently. Staff also noted the need to adapt practice and interventions for neurodivergent patients. Feedback also highlighted the need for internal gatekeeping to prevent oversaturation of the service and to avoid the service being used solely as an alternate pathway for quicker ADHD/autism diagnosis. Patient feedback (n=11) described benefits of validation, self-understanding, and stronger family relationships, praising the compassionate and personalised care received. Two patients highlighted the need for structured aftercare and follow-up.

**Conclusion:**

Findings suggest a particular need for neurodivergence assessments during the perinatal period. Patient and staff feedback consistently reported improved self-understanding and family relationships but highlighted post-diagnostic gaps in support and aftercare, underlining areas for service improvement. This evaluation has highlighted the need for further development of the pathway for neurodivergence within the perinatal population ensuring the service provided is sustainable and adapted to meet patients’ needs.

